# Mucus-filled lesion of a distal viable remnant tip of an appendix that developed 23 years after appendectomy

**DOI:** 10.1016/j.ijscr.2020.05.070

**Published:** 2020-06-06

**Authors:** Hidenori Tomida, Shinichi Hashimoto, Masahiro Hayashi, Masamichi Koyama

**Affiliations:** a Department of General Surgery, Asama Nanroku Komoro Medical Center, 3-3-21 Aioicho, Komoro, Nagano, 384-8588, Japan; b Department of Pathology, Asama Nanroku Komoro Medical Center, 3-3-21 Aioicho, Komoro, Nagano, 384-8588, Japan

**Keywords:** CEA, carcinoembryonic antigen, CA19-9, cancer antigen 19–9, PAML, post-appendectomy mucus-filled lesion, Appendix, Post-appendectomy mucus-filled lesions, Appendiceal stump, Distal viable remnant tip of appendix, Incomplete appendectomy, Surgical complication

## Abstract

**Introduction:**

Post-appendectomy mucus-filled lesions, arising from appendiceal remnant tissue, are an exclusively rare clinical entity. We report a rare case of a mucus-filled lesion rising from a distal viable remnant appendiceal tip as a late complication of appendectomy.

**Presentation of case:**

A 48-year-old man presented with a 2-week history of right buttock and right lower quadrant abdominal pain. He had previously undergone appendectomy 23 years ago. Radiological examination showed a cystic lesion lateral to the colon with a prominent tubular structure. According to the past operative note, the appendiceal tip was lost accidentally. Surgical resection was performed. Pathologic examination revealed a mucus-filled lesion lateral to the cecum, with no communication between them. There was no evidence of malignancy. The prominent structure had muscular layer, suggestive of distal appendiceal remnant.

**Discussion:**

Surgical resection of appendiceal mucus-filled lesion is recommended, because there are no reliable criteria to exclude malignant lesions. Although appendiceal mucus-filled lesion is rare, it is extremely rare in patients with previous appendectomy. In our case, the mucinous material secreted from the remnant appendix might be enclosed by the surrounding tissue.

**Conclusion:**

In patient with previous appendectomy who show a cystic mass close to the cecum, the possibility of lesion derived from the appendiceal stump or distal appendiceal remnant should be considered. In such cases, it is important to review the medical records for a definitive diagnosis. Complete appendectomy, without leaving appendiceal tissue, remains mandatory to prevent the development of late complications of mucus-filled lesions.

## Highlights

• Appendectomy is currently the most common surgical operation worldwide.
• Post-appendectomy mucus-filled lesions are a rare clinical phenomenon.
• It is vital to perform appendectomy completely, without leaving appendiceal tissue.
• This prevents late complication of mucus-filled lesion formation.

## 1 Introduction

Mucocele is a rare clinical phenomenon of appendiceal mucus-filled lesion. Preoperative diagnosis is important to prevent intraoperative perforation, which may result in pseudomyxoma peritonei [[1], [2], [3]]. Appendiceal mucocele, while uncommon and hard to be detected in the context of previous appendectomy, has been described [[4], [5], [6]]. In contrast, a mucus-filled lesion derived from a distal viable leftover remnant tip of the appendix following incomplete appendectomy has not.

We present a rare case of mucus-filled lesion derived from a viable leftover appendiceal tip as a late complication of open appendectomy.

The work has been in line with the SCARE criteria 7

## 2 Presentation of case

An active 48-year-old man presented with a 2-week history of right buttock and sudden right lower quadrant (RLQ) abdominal pain. He had normal appetite and bowel habits. His past medical history was unremarkable except for an acute appendicitis and open appendectomy 23 years prior to this visit. His body weight, height, and body mass index were 95.2 kg, 178.0 cm and 30.0, respectively. He was a hotel worker and had a cigarette smoking history of one pack a day. There was no family history of malignancies.

At presentation, his vital signs were stable. Physical examination was remarkable for a soft, nondistended abdomen with tenderness in the right lateral to lower abdomen with past appendectomy scar. There was no rebound tenderness or guarding. An emergent condition was suspected, including diverticulitis, ileitis, and enteritis.

Serum tumor markers and cancer antigen 19–9 (CA19-9 2.2 U/mL) were within normal range; however, carcinoembryonic antigen (CEA; 8.2 ng/mL) levels were elevated.

**Fig. 1:**
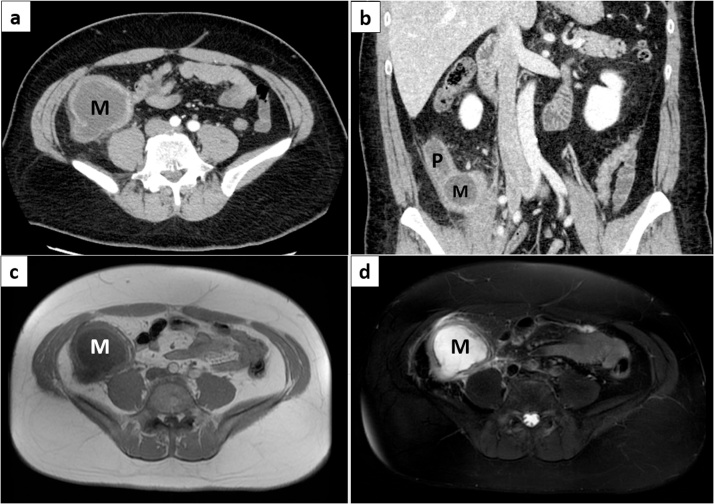
Contrast-enhanced computed tomography image revealing a hypodense cystic mass lesion lateral to the caecum, not communicating with the lumen. The lesion has a weakly enhancing wall with scattered mural calcification and a prominent structure located along the retroperitoneum. In axial view (a) and in coronal view (b). Magnetic resonance imaging revealing a cystic mass lesion that was markedly hypointense on T1-weighted images (c) and hyperintense on T2-weighted images (d). M: cystic mass, P: prominent structure.

Abdominal contrast-enhanced computed tomography showed a well-defined hypodense (15–20 Hounsfield units) cystic mass lesion measuring approximately 7.0 × 12.0 cm lateral to the cecum, which appeared not to be communicating (Fig. 1). The lesion’s border was adjacent to the retroperitoneum, iliopsoas muscle, and right urinary duct, suggesting inflammation or invasion. The lesion had a weakly enhanced and thickened wall with scattered mural calcification, and a prominent tubular structure located along the retroperitoneum.

Magnetic resonance imaging showed a well-circumscribed lesion that was markedly hypointense and hyperintense on T1- and T2-weighted images, respectively (Fig. 1).

Colonoscopic findings were normal; however, the appendiceal orifice could not be identified.

**Fig. 2:**
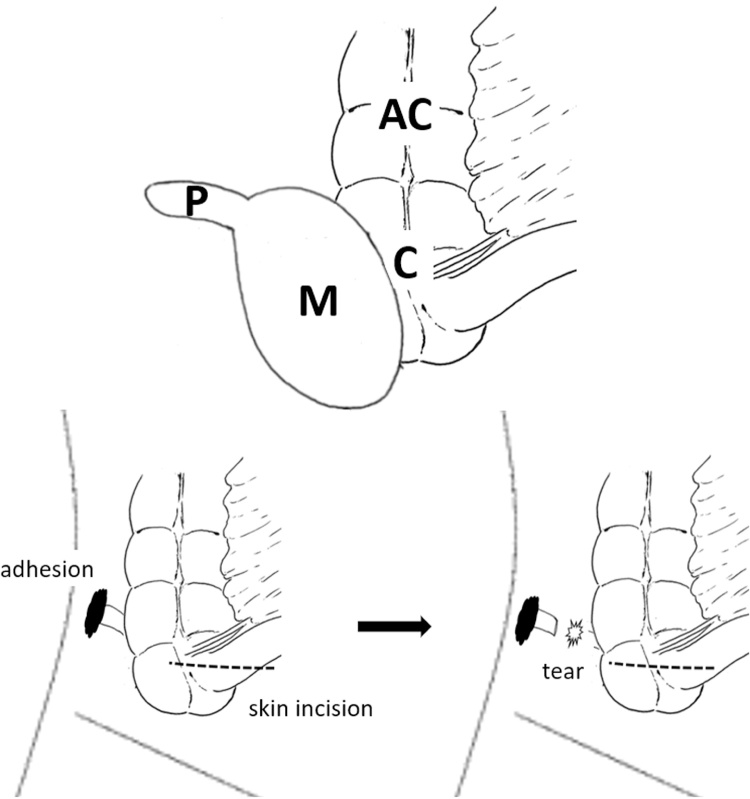
Schematic illustration of the ileocecal lesion and prior open appendectomy. The cystic mass with a prominent structure. M: cystic mass, P: prominent structure, C: caecum, AC: ascending colon.

Schematic illustration of the expected ileocecal lesion is shown (Fig. 2).

Past operative note revealed that his past open appendectomy 23 years prior had several issues. The spinal anesthetic effect was not stable, and skin incision was done closer to the midline than usual. The appendix was found to have a long body with the distal tip strongly adhered to the retroperitoneum; therefore, retrograde appendectomy was performed. During tissue peeling, the appendix accidentally detached, and the tip was lost (Fig. 2). Despite extending the skin incision, the tip could not be found. The operation was discontinued once the anesthesia wore off.

Histopathologic examination revealed phlegmonous appendicitis without any malignancy. Postoperatively, he complained of RLQ abdominal pain, and computed tomography (CT) showed inflammation of the periphery of ileocecum at 2 weeks after surgery. His symptoms resolved transiently after antibiotic therapy.

Considering the possibility of infectious diseases, the patient received empirically intravenous antibiotics (piperacillin/tazobactam); however, his abdominal pain and inflammatory markers did not improve.

Conducting elective exploratory laparotomy with preceding ureteral stent placement found a well-encapsulated mass, adherent to the surrounding tissues lateral to the cecum, which had a prominent structure similar to a distal appendiceal remnant. The wall was mildly inflamed, but there was no evidence of perforation or abscess formation. There was no lymph node enlargement, and the mass was not easily mobilized from adjacent structures. Therefore, en bloc ileocecal resection with sufficient margins and systematic lymph node dissection were performed.

**Fig. 3:**
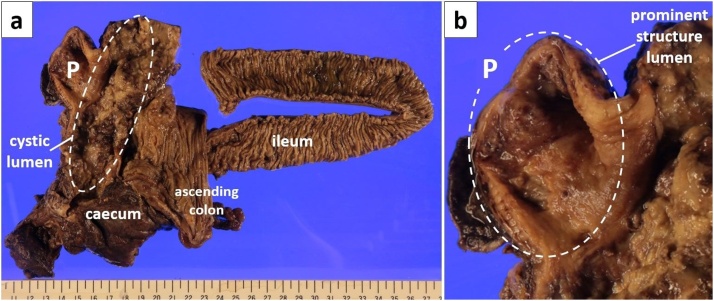
Macroscopic view of the opened ileum, caecum, cystic mass lesion (a). It revealed a distended mucus-filled cystic mass lateral to the caecum with no communication with the lumen. The lesion had a prominent structure (b). P: prominent structure.

**Fig. 4:**
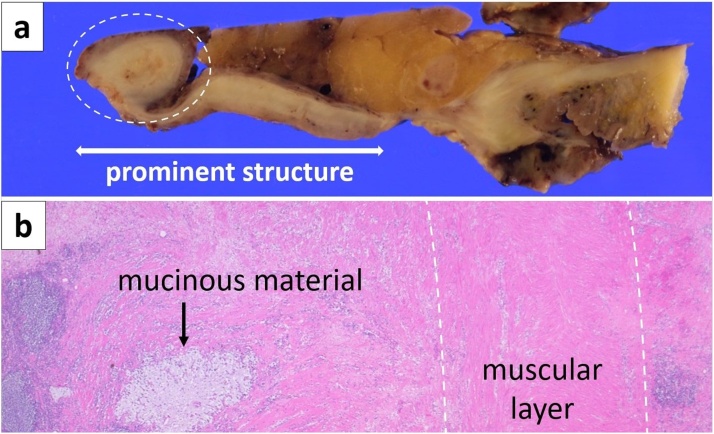
Macroscopic view of the long-axis cross section of the opened prominent structure (a). Low power microscopic view of the prominent structure tip revealed mucinous material and surrounding muscular layer (b). Mucosa layer was almost lost; however, the muscular layer was confirmed over the entire prominent structure surface.

Gross pathologic examination revealed a distended mucus-filled lesion lateral to the cecum without communicating with the lumen (Fig. 3). The mucinous material contained a sand-like calcified component. The prominent tubular structure appeared similar to a distal appendiceal remnant filled with mucinous materials. On histopathological examination, the mass had a non-epithelial wall and there was no evidence of adenoma or adenocarcinoma. The wall had partial mural calcification, suggestive of long-term minimal inflammation. Mesenteric lymph nodes were free of pathological involvement. Mucinous material was adjacent to the muscular layer of the cecum, and the serosal layer could not be identified. The prominent structure had almost no mucosa layer; however, the muscular layer remained, suggestive of distal appendiceal remnant (Fig. 4).

**Fig. 5:**
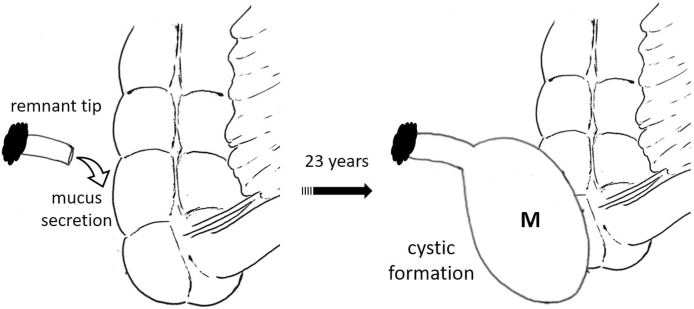
Schematic illustration of mucus-filled cystic mass formation. Distal remnant of appendiceal tip maintained its vascularization and secreted mucus continuously, leading to cystic formation. M: cystic mass.

The patient was diagnosed with a mucus-filled lesion rose from the distal viable remnant tip of the appendix (Fig. 5). Perioperative antibiotics was given and culture tests were negative.

The patient’s postoperative recovery was uneventful. He was symptom-free and maintained a normal CEA level (2.2 ng/mL) during the 4-month follow-up period.

## 3 Discussion

Appendiceal mucus-filled lesion, called mucocele, is a rare clinical entity first described in 1842 [8]. It denotes a proximal obstructive dilatation of the appendiceal lumen, usually communicating with the cecum, by viscous mucoid/mucinous material. The causes of obstruction include fecality, hyperplasia, tumor, post-inflammatory fibrosis, endometriosis, and developmental anomalies [9]. In 2012, the Peritoneal Surface Oncology Group International (PSOGI) developed a consensus classification [10].

Clinical presentations of appendiceal mucocele are nonspecific, including abdominal pain with appendicitis, nausea and vomiting, weight loss, palpable mass, changes in bowel habits, or lower gastrointestinal bleeding; [10] for these reasons, the rate of accurate preoperative diagnosis is low.

Preoperative diagnosis is not only important to prevent perforation, which may result in pseudomyxoma peritonei [[1], [2], [3]], but also understand differential nature of appendiceal mucocele [9,12].

Surgical resection of all appendiceal mucinous lesions is recommended for both diagnostic and therapeutic purposes, because there are no reliable criteria to exclude malignant lesions [1,[13], [14], [15]]. An algorithm for the selection of surgical type has been formulated by Dhage-Ivatury and Sugarbaker [16]. The treatment of appendiceal mucocele is simple appendectomy given the presence of a normal cecum and appendicular base without perforation.

During spontaneous perforation, leakage from the cyst, enlarged mesenteric lymph node, or positive cytology strongly suggestive of malignancy, right hemicolectomy should always be performed to achieve complete cure [1,13].

The RLQ abdominal pain in adults who have undergone appendectomy represents a diagnostic challenge. The WISS study confirmed acute appendicitis as the most frequent cause of intra-abdominal infection, [17] and appendectomy is currently the most common surgical operation worldwide.^18^

Stump appendicitis (SA) is a rare case of inflammation of remnant appendiceal tissues due to the incomplete removal of the appendix [19,20], Although many causative agents of SA have been described, the main factor remains the length of remnant appendix, where a >25-mm length represents a possible reservoir for fecalith and results in inflammation [18,19]. By contrast, the appendiceal stump, which atypically develops into a mucus-filled lesion stump mucocele (SM), can cause pseudomyxoma peritonei [21]. Additionally, appendiceal mucocele is extremely uncommon in a patient with a previous appendectomy [5,16].

Nine cases of post-appendectomy mucus-filled lesion (PAML) were reported (Table 1). As previously described the time interval between the initial operation and re-operation of PAML is longer than that of SA [22].

**Table 1:** Cases of mucus-filled lesion in a patient with post-appendectomy.

No	Author	year	Age	Gender	Main symptoms	Interval time year	Initial operation age	Size (cm)	Origin	Histopathology
1	W. Lien	2004	66	M	RLQ pain	30	36	8	Stump	mucinous cystadenoma
2	W. Lien	2004	45	F	RLQ pain	10	35	ND	Stump	ND
3	D. Korkolis	2006	49	F	RLQ pain	25	24	8	Stump	mucinous cystadenoma
4	M. Johnson	2006	67	M	RLQ pain	15	52	6	Distal	mucinous cystadenoma
5	N Creuzé	2008	45	M	Abdominal pain	16	29	ND	Stump	simple mucocele
6	M. El Ajmi	2009	54	F	RLQ pain	20	34	90	Stump	mucinous cystadenoma
7	J. Cama	2010	27	F	RLQ pain	18	9	3	Stump	simple mucocele
8	M. Kim	2010	78	M	Palpable mass	40	38	Huge	Stump	mucinous cystadenoma
9	A Ozgür	2012	75	M	Abdominal pain	10	65	9	Stump	mucinous adenocarcinoma
10	Our case	2020	48	M	RLQ pain	23	25	12	Distal	mucus-filled lesion

M: Male, F: Female, RLQ pain: Right Lower Quadrant pain, Stump: Appendiceal Stump, Distal: Distal viable remnant tip of appendix, ND: Not described.

Compared to Johnson et al.’s study, ^6^ in which the lesions were also derived from the distal viable remnant tip, our case had a non-epithelial wall. Similar to SA, the mechanism of PAML formation is likely due to incomplete removal of the appendix [23]. Here, the distal remnant of the appendiceal tip is presumed to maintain its vascularization, leading to the formation of a mucus-filled lesion [6]. In our case, the mucinous material secreted from the remnant appendix might be enclosed by the mesentery, peritoneum, retroperitoneum, and cecum serosa. While the cause of abdominal pain remained unknown, it is assumed that inflammation, such as infection or chemical reaction, was induced because of gastrointestinal infection or mucinous ingredient.

Since an unusual case of simple mucocele with elevated serum CEA had been reported, [24] considering that CEA level normalized after surgical excision, the lesion described here probably had high level of CEA despite not displaying malignancy.

## 4 Conclusion

Ultimately, in patient with previous appendectomy who showed a cystic mass close to the cecum, not connected to the lumen, one should take the possibility of PAML derived from appendiceal stump or distal viable remnant appendiceal tip into consideration.

It is also important to review the histology of the appendix and previous operative notes for definitive diagnosis. The surgical treatment of acute appendicitis has undergone a paradigm shift from open to laparoscopic surgery. However, complete appendectomy, without leaving appendiceal tissue, remains mandatory to prevent complications of mucus-filled lesions.

## Declaration of Competing Interest

None declared.

## Sources of funding

None

## Ethical approval

Ethical approval has been exempted by my institution.

## Consent

Written informed consent was obtained from the patient for publication of this case report and accompanying images. A copy of the written consent is available for review by the Editor-in-Chief of this journal on request.

## Author contribution

HT wrote the draft of the manuscript. SH revised the article. HT and SH performed perioperative care and performed the operation. MK is the pathologist. All authors participated in the management of the patients in this case report.

## Registration of research studies

Not applicable.

## Guarantor

Shinichi Hashimoto.

## Provenance and peer review

Not commissioned, externally peer-reviewed.
